# Feasibility of digital multidomain cognitive testing in mild cognitive impairment and dementia assessment in primary care

**DOI:** 10.1177/25424823261492238

**Published:** 2026-10-07

**Authors:** Wobbie Van den Hurk, Maria M. Johansson, Elisabet Classon, Anders Gustavsson, Katarina Nägga, Anna Segernäs

**Affiliations:** 1 Department of Health, Medicine and Caring Sciences, 4566 Linköping University, Linköping, Sweden; 2 Mindmore AB, Stockholm, Sweden; 3 Department of Activity and Health, Region Östergötland, Linköping, Sweden; 4 Department of Geriatrics and Palliative Medicine, Region Östergötland, Linköping, Sweden; 5 526215 Quantify Research, Stockholm, Sweden; 6 Primary Health Care Centre in Ekholmen, 4564 Region Östergötland, Linköping, Sweden

**Keywords:** Alzheimer's disease, digital technology, early diagnosis, feasibility studies, neurocognitive disorders, neuropsychological tests, primary health care, user-computer interface

## Abstract

**Background:**

Primary care plays a central role in identifying and monitoring mild cognitive impairment (MCI), Alzheimer's disease and other dementias, yet access to suitable cognitive assessment tools remains limited.

**Objective:**

To evaluate the feasibility, acceptability, usability and preliminary clinical validity of a digital multidomain cognitive test battery in routine primary care neurocognitive assessments.

**Methods:**

A total of 77 patients undergoing neurocognitive assessment across eight Swedish primary care centers were recruited and grouped by final diagnosis: 26 subjective cognitive impairment (SCI), 28 MCI, and 23 dementia. Participants completed a digital cognitive test battery consisting of eight traditional tests administered on the Mindmore platform via touchscreen tablets and supervised by occupational therapists (OTs). Clinicians were blinded to test results. Cognitive performance was compared across diagnostic groups and against normative data. Patient and OT experiences were collected using structured surveys.

**Results:**

Cognitive performance declined progressively from SCI to MCI to dementia on all tests. The battery yielded a detailed cognitive profile spanning 10 main test results across five cognitive domains. All included patients completed the test battery, they reported clear instructions, and 58% preferred tablet-based over paper-based testing. OTs considered the battery appropriately challenging in this population, provided some assistance in 69% of cases, and expected the battery to yield reliable results.

**Conclusions:**

These findings support the feasibility of integrating multidomain digital cognitive testing in primary health care with limited clinician burden. This is a promising approach for increasing availability of standardized detailed cognitive assessments at early levels of care.

## Introduction

Early and accurate identification, characterization, and follow-up of neurocognitive disorders have become increasingly important with the emergence of disease-modifying treatments and structured non-pharmacological interventions.^1–3^ With population ageing, the prevalence of dementia and mild cognitive impairment (MCI) continues to rise globally, increasing demands on health systems and primary care services.^4,5^

Primary health care (PHC) is typically the first point of contact for individuals experiencing cognitive change and plays a central role in both the assessment and management of neurocognitive disorders.^6,7^ Ideally, PHC assessments detect the disorders at the earliest stages, exclude alternative causes, support differential diagnosis, and guide appropriate treatment, counseling, and follow-up, and only refer the most complex cases to specialist care. This is challenging due to the heterogeneous causes of cognitive symptoms, including Alzheimer's disease (AD), vascular pathology, Lewy body disease (LBD), and frontotemporal degeneration (FTD), which also frequently co-occur.^8,9^ In addition, patients may present non-neurocognitive conditions which independently impair cognition such as depression^10^ and stress-related disorders.^11^ Furthermore, patients seek assessment across the full disease spectrum, from subjective cognitive impairment (SCI) to established dementia, adding substantial heterogeneity in symptom severity.

Recent advances in fluid biomarkers, particularly in blood-based biomarkers (BBM) for AD and FTD, offer new possibilities for identifying underlying pathology in these cases, and are likely to become accessible in PHC in the future.^12,13^ However, biomarkers do not establish the symptomatic cause of cognitive complaints, as pathological changes may precede clinical onset and mixed pathologies are common. Comprehensive clinical evaluation, including cognitive assessment across multiple domains, remains essential to interpret biomarker findings and determine clinical relevance.^6,14–16^ Instruments suitable for use in PHC, that provide information on the pattern and severity of impairment support differential diagnosis and detect subtle impairment in a way that brief screening instruments, often optimized for AD detection, cannot.

Digital cognitive test batteries provide standardized administration, often self-administrated, and automated scoring and comparison with normative data.^17–20^ Such tools can allow for multidomain cognitive profiling without unduly increasing clinician workload. A recent review found good feasibility of remote, unsupervised digital cognitive assessments in preclinical AD in research settings.^20^ Qualitative interviews with general practitioners (GPs), medical assistants, and patients also show generally positive attitudes towards digital cognitive testing in PHC.^17,21^ However, the populations in the studies were relatively healthy, did not span the full spectrum SCI to dementia, and were thus not representative for a real-world heterogeneous PHC population. In addition, most tools considered did not provide the type of detailed multidomain cognitive profile that may help establish type and severity of neurocognitive disorder.^22^

The Mindmore digital cognitive assessment platform, developed in Sweden, provides digital versions of 19 traditional cognitive tests and a range of clinically relevant scales, designed for self-administration. The platform, which runs on tablets and laptops, can be configured into batteries of varying length and domain coverage. As digital adaptations of established instruments, these tests are familiar to many clinicians and expected to largely inherit the extensive evidence base for clinical use in MCI and dementia.^23,24^

In this study, we selected a battery of eight cognitive tests spanning five domains: memory, attention and processing speed, executive functioning, visuospatial functioning, and language. This study aimed to evaluate the feasibility, acceptability, and usability of this multidomain test battery under supervised administration embedded within routine PHC neurocognitive assessments, with clinicians blinded to the test results to also explore the tests’ preliminary clinical validity across the neurocognitive disease spectrum.

## Methods

### Study design

This investigator-initiated, multicenter feasibility study was conducted at eight PHC centers (PHCC) in Östergötland, Sweden (2021–2024). Occupational therapists (OTs) identified eligible study participants based on the study's inclusion and exclusion criteria. All patients received standard PHC care, including routine cognitive assessment and referral to specialist services when indicated. In addition, participants completed a digital questionnaire and cognitive test battery using the Mindmore platform. Tests were administered on touchscreen tablets (Microsoft Surface Pro, 12.3 inches) and supervision by an OT in order to evaluate ability to perform digital testing in a population including individuals with severe cognitive impairment who might be unable to follow task instructions and maintain comprehension throughout the assessment. OTs were instructed to only provide assistance upon request or when necessary to ensure task completion.

Testing was typically conducted during home visits, either on the same occasion as the PHC assessment or within approximately two weeks. Clinicians remained blinded to all digital test results. Immediately after testing, patients and OTs completed hybrid surveys regarding their experience. Additional clinical information was obtained through GPs.

Ethical approval was granted by the Regional Ethics Review Board in Stockholm (2020-03390), and all participants provided written informed consent.

### Participants

A total of 80 participants were recruited: 31 from three PHCC in Norrköping and 49 from five PHCCs in Linköping. Recruitment was intended to be consecutive but was interrupted at times by increased clinical demands (e.g., during the COVID-19 pandemic). Inclusion criteria required that the patient was able to complete the cognitive screening tests used during the PHC neurocognitive assessment; was a fluent Swedish speaker; and, was able to comprehend the study information and provide informed consent. Exclusion criteria consisted of a previously established neurocognitive diagnosis; severe or unstable psychiatric disorder; traumatic brain injury or cerebral infarct within the past six months; ongoing alcohol use disorder; and motoric disabilities interfering with basic tablet interaction. Three recruited patients were subsequently excluded: two due to ongoing alcohol use disorder and one due to impaired manual dexterity following recent minor hand surgery.

### Diagnostic procedure

PHC neurocognitive assessment, initiated by a GP, included a systematic medical history, a medical examination (somatic, neurological, and psychiatric), the Cognitive Impairment Questionnaire (CIMP-QUEST),^25^ the Functional Activities Questionnaire completed by next of kin (FAQ),^26^ a computed tomography (CT) scan of the brain, and cognitive screening tests. These comprised the Mini-Mental State Examination (MMSE)^27^ and the Clock Drawing Test (CDT)^28^ per national guidelines,^16^ supplemented by the Neurobehavioral Cognitive Status Examination (NCSE)^29^ and the Alzheimer's Quick Test (AQT)^30^ per regional clinical practice in Linköping. Clinical diagnoses were established in PHC through structured team evaluation involving a GP, nurse and OT, with diagnostic support from a consultant geriatrician when needed, following clinical practice and usual care in the central part of Region Östergötland, Sweden.

When diagnostic uncertainty remained, patients were referred to a specialist memory clinic. At the memory clinic, patients underwent extended cognitive testing, in-depth evaluations by geriatric specialists, and when relevant, neuropsychological evaluation, Magnetic Resonance Imaging (MRI) of the brain and cerebrospinal fluid (CSF) biomarker analysis. For the purpose of this study, all available data collected within six-months after the digital cognitive assessment, as well as information already available from medical records, was considered when determining the final diagnosis. In addition, patients with suspected normal pressure hydrocephalus (NPH) were referred to a specialist neurology department; in these cases, the neurocognitive PHC diagnoses were retained in the study dataset.

Final diagnoses were determined through joint review of all available clinical data by a geriatric specialist and the diagnosing clinicians, and DSM-5 criteria^31^ were systematically applied to confirm diagnoses of MCI and dementia to mild and major neurocognitive disorders, respectively. The label SCI was applied when patients reported cognitive decline without corresponding impairment in the PHC neurocognitive assessment. The geriatric specialist and the diagnosing clinicians remained blinded to digital test results.

### Digital cognitive assessment

The selected test battery covered the domains memory (10-word CERAD Word List Learning Test),^32^ attention and processing speed (Trail Making Test [TMT] Part A^33^ and Symbol Digit Processing Test [SDPT]),^34^ executive functions (TMT Part B),^33^ visuospatial functions (Cube Copying Test^35^ and Clock Drawing Test),^28^ and language (Token Test^36,37^ and Boston Naming Test [BNT]).^38,39^

The tests were administered in a fixed order: TMT A, TMT B, CERAD Learning Trials 1–3, Cube Copying Test, Clock Drawing Test, CERAD Delayed Recall, CERAD Recognition, SDPT, Token Test, and BNT. Administration was standardized and automated as designed by Mindmore, with audio and written instructions, repeatable on demand, and for this study conducted under supervision by an experienced OT. TMT A, TMT B, and SDPT were preceded by a practice trial, unsuccessful practice (maximum five attempts) led to the test being omitted. Results for omitted tests were imputed with the minimum result present in the current data. Detailed test descriptions and scoring procedures are provided in the Supplemental Methods.

Previous research on the Mindmore platform has demonstrated comparability with paper-based test administration,^40^ adequate test–retest reliability,^41^ convergent validity with conventional neuropsychological measures,^42^ and good usability in both supervised and unsupervised settings in healthy^42,43^ and perioperative participants.^44^ Test results are automatically scored and standardized against platform-specific normative data from the cognitively healthy Swedish population.^45,46^

### Quantitative analyses

Summary statistics were generated for all participants for both the PHC neurocognitive assessment and the digital cognitive assessment. Group differences were examined using *t*-tests, χ^2^ tests, Fisher's exact tests, and one-way ANOVAs with appropriate post hoc comparisons.

Expected digital cognitive test scores were estimated for a demographically matched group of “normative controls” to reflect the clinical routine of comparing patient results against normative data. The normative controls were generated using the regression-based models generated from the platform-specific normative sample of 1297 cognitively healthy Swedish adults (aged 18–93) who completed a Mindmore test battery.^45,46^ This approach is inspired by the virtual controls method used in randomized clinical trials^47^ and previously applied to remote digital cognitive testing in stress-related disorders.^48^ One model was fitted per test result, accounting for age, education, sex and input device (touchscreen, mouse or touchpad), with linear, non-linear and interaction terms retained where they significantly contributed to variation. For each patient, expected scores were derived from these models using the patient's age, sex, and education, with touchscreen as input device. To capture natural variance not explained by these variables, 100 normative controls per patient were sampled from the normal distribution around each expected score, using the standard deviation (SD) of the model residuals from the normative sample. This efficiently provides each patient with a demographically matched normative comparison without recruiting a separate control group, although the normative controls are model-derived and therefore inherit the assumptions and limitations of the normative models rather than representing an independently observed sample. All analyses were performed in R 4.4.1.^49^

### Qualitative and survey-based analysis

Patient and OT experiences were evaluated using hybrid surveys combining multiple-choice and open-ended questions, completed directly after digital test administration. Patients and OTs were oblivious to the digital test results. Content analysis (inspired by Bengtsson)^50^ was used to analyze the qualitative responses on the questions: “Can you [patient] describe whether you experienced any differences between completing the tests on a tablet and the previous pen-and-paper testing [typically MMSE and CDT]?” and “Did you [OT] need to provide any assistance during testing. If so, what was it related to?”. Note that patient comparisons between test formats may have been affected by the time lapse between traditional and digital testing. Categories were developed inductively, and responses were coded to indicate endorsement of each category.

## Results

### Sample characteristics and PHC neurocognitive assessment

The final sample included 77 participants (Table 1). As expected, the sample showed large variance in terms of medical history and symptom severity. Final neurocognitive diagnoses were: SCI (n = 26), MCI (n = 28; eight AD, three vascular, one mixed, 10 unspecified, four attributed to possible NPH, and two to possible stress-related disorders), and dementia (n = 23; 16 AD late onset, five mixed dementia, and two unspecified dementia).

**Table 1.** Final sample demographics and PHC neurocognitive assessment results by diagnostic group.

	SCI (n = 26)	MCI (n = 28)	Dementia (n = 23)	Total (N = 77)
Demographics				
Age, years	68.2 ± 11.9	73.8 ± 9.7	79.1 ± 6.8	73.5 ± 10.6
	(41–85)	(48–90)	(63–90)	(41–90)
Male, n (%)	14 (54%)	15 (54%)	11 (48%)	40 (52%)
Female, n (%)	12 (46%)	13 (46%)	12 (52%)	37 (48%)
Education Level, n (%)^a^				
- Primary (7 years)	4 (15%)	8 (29%)	8 (35%)	20 (26%)
- Secondary (11 years)	8 (31%)	8 (29%)	9 (39%)	25 (32%)
- Tertiary (15 years)	14 (54%)	12 (43%)	6 (26%)	32 (42%)
Living alone, n (%)	7 (27%)	9 (32%)	8 (35%)	24 (31%)
Currently employed, n (%)	7 (27%)	3 (11%)	0 (0%)	10 (13%)
Native Swedish speakers, n (%)	22 (85%)	27 (96%)	23 (100%)	72 (94%)
Medical History, n (%)				
Ischemic heart disease	6 (23%)	5 (18%)	4 (17%)	15 (19%)
Atrial fibrillation	5 (19%)	3 (11%)	4 (17%)	12 (16%)
Hypertension	10 (38%)	14 (50%)	12 (52%)	36 (47%)
Diabetes Mellitus	5 (19%)	3 (11%)	5 (22%)	13 (17%)
Hypothyroidism^b^	2 (8%)	3 (11%)	0 (0%)	5 (6%)
Cerebrovascular insult	2 (8%)	5 (18%)	1 (4%)	8 (10%)
Mental illness	5 (19%)	5 (18%)	2 (9%)	12 (16%)
Neurological disease	4 (15%)	5 (18%)	3 (13%)	12 (16%)
PHC neurocognitive assessment				
MMSE	28.42 ± 1.65	27.46 ± 1.79	24.39 ± 3.13	26.87 ± 2.77
	(24–30)	(23–30)	(19–29)	(19–30)
CDT, correct (%)	25 (96%)	23 (82%)	15 (65%)	63 (82%)
CDT, incorrect (%)	1 (4%)	5 (18%)	8 (35%)	14 (18%)
FAQ	1.71 ± 3.26	3.24 ± 3.36	9.42 ± 6.29	4.43 ± 5.35
	(0–14)	(0–13)	(0–25)	(0–25)
CIMP-QUEST				
- Temporoparietal symptoms	2.00 ± 2.33	2.77 ± 2.96	6.22 ± 3.69	3.58 ± 3.49
	(0–8)	(0–11)	(0–16)	(0–16)
- Frontal symptoms	2.24 ± 2.22	3.35 ± 3.57	6.83 ± 4.69	4.05 ± 4.04
	(0–7)	(0–16)	(1–18)	(0–18)
- Subcortical symptoms	4.28 ± 4.44	3.19 ± 3.81	6.70 ± 5.99	4.65 ± 4.94
	(0–15)	(0–15)	(0–20)	(0–20)
- Associated (psychiatric) symptoms	3.12 ± 2.40	3.81 ± 3.56	5.52 ± 3.45	4.11 ± 3.29
	(0–8)	(0–11)	(1–15)	(0–15)
- Memory symptoms	3.28 ± 2.25	4.88 ± 3.05	8.48 ± 3.15	5.46 ± 3.53
	(0–8)	(0–12)	(2–14)	(0–14)

Results are reported as mean ± SD (standard deviation) and (range) unless otherwise specified. ^a^Primary, secondary, and tertiary education refers to the Swedish folkskola/grundskola, realskola/gymnasium, universitet/högskola, respectively. ^b^No participant had hyperthyroidism. Results are missing for: 9x FAQ and 3x CIMP-QUEST. SCI: subjective cognitive impairment; MCI: mild cognitive impairment; MMSE: Mini-Mental State Examination; CDT: Clock Drawing Test; FAQ: Functional Activities Questionnaire; CIMP-QUEST: Cognitive Impairment Questionnaire. Both the FAQ and CIMP-QUEST were completed by next of kin.

Participants with SCI were significantly younger than those with dementia (F(2, 74) = 7.535, *p* = 0.001), whereas age did not differ significantly between the MCI group and either SCI or dementia. No significant group differences were observed in sex distribution or education level. All but five participants were native Swedish speakers. The non-native speakers all spoke and understood Swedish fluently and had lived in Sweden for a median of 31 years (IQR = 12–33; interquartile range, 25^th^–75^th^ percentile).

Across PHC screening tests, participants with dementia had significantly lower MMSE and FAQ scores than those with SCI or MCI, who did not differ from one another (MMSE: F(2, 74) = 21.346, *p* < 0.001; FAQ: F(2, 65) = 18.166, *p* < 0.001). CDT performance differed between groups (Fisher's Exact test, *p* = 0.018), although post hoc comparisons did not reach significance following Holm correction.

Results from the CIMP-QUEST showed significant group differences in all symptom domains included. Participants with dementia showed significantly more temporoparietal (F(2, 71) = 13.109, *p* < 0.001), frontal (F(2, 71) = 10.500, *p* < 0.001), and memory-related symptoms (F(2, 71) = 20.933, *p* < 0.001) than those with SCI or MCI. Subcortical symptoms were elevated in dementia relative to MCI (F(2, 71) = 3.387, *p* = 0.039) and associated (psychiatric) symptoms were higher in dementia compared with SCI (F(2, 71) = 3.603, *p* = 0.032).

Twenty patients were referred to a memory clinic for further examination (final diagnoses: four SCI, nine MCI, and seven dementia). Of these, 11 had not received a preliminary diagnosis in PHC. Among the nine who had, only one diagnosis changed at the memory clinic (from SCI to MCI); the remaining preliminary diagnoses (two SCI, three MCI, and three dementia) were confirmed.

### Timing

The diagnostic process in PHC spanned several months. A median of 84 days passed between the PHC cognitive assessment, performed during a home visit, and the final diagnostic decision (IQR = 41.5–181). Digital cognitive testing occurred on or close to this home visit, with a median of 77 days between test completion and final diagnosis (IQR = 28.5–178).

### Test results for the digital cognitive assessment

All included participants completed the cognitive test battery from start to finish. Median test administration time was 43.5 min (IQR = 37–49.2), varying slightly by diagnostic group (SCI: 38 min; MCI: 44 min; dementia: 48 min). In total, 14 single tests were omitted from administration due to repeated failure on the practice trial: 1x SDPT (MCI: 1); 1x TMT A (SCI: 1); 12x TMT B (SCI: 2, MCI: 5, dementia: 5). These results were imputed with the worst obtained score in the sample: SDPT correct: 1; TMT A Time to completion: 120 s; TMT A Correct connections: 15; TMT B Time to completion: 240 s; TMT B Correct connections: 0. Acknowledging this might overestimate patient performance, specifically for TMT B which has known floor effects in this population.^51^ One participant's Token Test result was invalid (MCI: 1) and detailed Cube and Clock metrics were unavailable for 35 participants, as these measures were introduced part-way through the study.

Across test results, the dementia group showed the lowest performance, followed by the MCI group, while the SCI group performed similarly to or somewhat below the norms but better than the other two clinical groups (Table 2 and Figure 1). The tests differentiated most clearly between groups in the memory domain (Figure 1(a)–(c)), with differences of around one SD between the SCI and MCI groups, and a comparable separation between the MCI and dementia groups. The tests also significantly differentiated the MCI group from the norms or SCI group in the domains of attention and processing speed (Figure 1(d) and (e)), executive functions (Figure 1(f)), and language (Figure 1(i) and (j)). In the visuospatial functions domain (Figure 1(g) and (h)), significant differentiation was observed only between the dementia and norms or SCI group.

**Figure 1.**
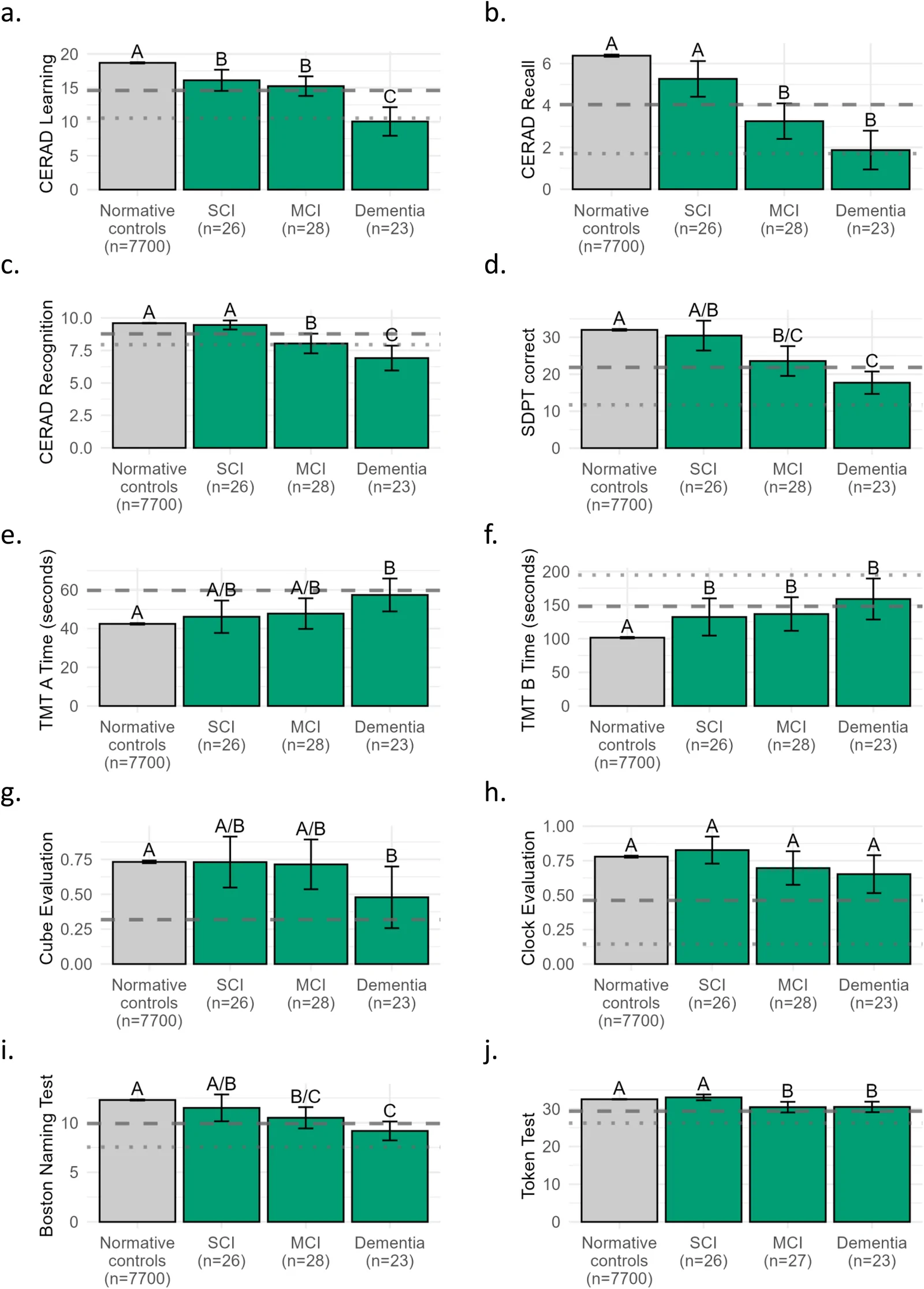
Test results of the digital cognitive assessment. Cognitive domains and functions: a. Memory - Verbal learning; b. Memory - Recall; c. Memory - Recognition; d. Attention and processing speed - Visual scanning; e. Attention and processing speed - Mental speed; f. Executive functions - Mental flexibility; g. Visuospatial functions - Cube Copying Test; h. Visuospatial functions - Clock Drawing Test; i. Language - Naming; j. Language - Auditory comprehension. Green bars represent patient performance; grey bars represent model-based normative controls generated using Mindmore's normative models.^45,46^ Error bars indicate 95% CI. Letter labels (A, B, C) denote post hoc pairwise comparisons: groups sharing a letter do not differ significantly, while groups with different letters differ significantly (Tukey-adjusted *p* < 0.05). Dashed and dotted grey lines indicate 1 and 2 SD below the normative control mean, or above it for TMT A and TMT B time to completion, where higher scores indicate poorer performance. The 2 SD line falls outside the plotted range in panels e and g. SCI: subjective cognitive impairment; MCI: mild cognitive impairment.

**Table 2.** Digital cognitive test performance by diagnostic patient group and the model-based normative control group.

Cognitive domain and test result	Normative controls (n = 7700)	SCI (n = 26)	MCI (n = 28)	Dementia (n = 23)	Group differences
Memory					
CERAD Learning	18.69 ± 4.08	16.12 ± 3.86	15.25 ± 3.72	10.04 ± 4.87	44.220***
	(5–30)	(10–23)	(7–22)	(0–18)	NC > SCI/MCI > D
CERAD Recall	6.38 ± 2.34	5.27 ± 2.11	3.25 ± 2.19	1.87 ± 2.14	46.880***
	(0–10)	(0–9)	(0–9)	(0–7)	NC/SCI > MCI/D
CERAD Recognition	9.60 ± 0.83	9.46 ± 0.86	8.04 ± 1.95	6.91 ± 2.21	110.025***
	(7–10)	(7–10)	(1–10)	(2–10)	NC/SCI > MCI > D
Attention & Processing Speed					
SDPT correct	32.01 ± 10.16	30.46 ± 10.00	23.57 ± 10.39	17.70 ± 7.01	21.735***
	(0–73)	(8–49)	(1–40)	(1–30)	NC > MCI, NC/SCI > D
TMT A Time to completion (seconds)	42.44 ± 17.32	46.12 ± 20.71	47.75 ± 20.41	57.39 ± 19.66	6.906***
	(1–120)	(25–120)	(23–120)	(29–110)	NC < D
TMT A Correct connections	23.84 ± 0.61	23.38 ± 1.83	23.64 ± 1.70	23.65 ± 0.83	6.351***
	(22–24)	(15–24)	(15–24)	(21 −24)	NC > SCI
Executive functions					
TMT B Time to completion (seconds)	101.50 ± 46.59	132.23 ± 68.36	136.61 ± 64.35	158.91 ± 70.45	20.309***
	(1–240)	(55–240)	(50–240)	(32–240)	NC < SCI/MCI/D
TMT B Correct connections	20.04 ± 6.13	19.54 ± 7.27	15.04 ± 9.54	12.04 ± 10.16	19.008***
	(0–24)	(0–24)	(0–24)	(0–24)	NC /SCI > MCI/D
Visuospatial functions					
Cube Copying Test Evaluation	0.73 ± 0.41	0.73 ± 0.45	0.71 ± 0.46	0.48 ± 0.51	2.877*
	(0–1)	(0–1)	(0–1)	(0–1)	NC > D
Cube Copying Test Time to completion (seconds)		62.05 ± 32.75	81.26 ± 44.19	146.98 ± 126.93	4.690*
		(16–126)	(35–166)	(44–388)	SCI < D
Cube Copying Test Clicked erase		0.65 ± 0.86	1.00 ± 1.76	1.85 ± 2.64	1.63
		(0–3)	(0–6)	(0–9)	
Clock Drawing Test Evaluation	0.78 ± 0.32	0.83 ± 0.24	0.70 ± 0.31	0.65 ± 0.32	2.057
	(0–1)	(0.5–1)	(0–1)	(0–1)	
Clock Drawing Test Time to completion (seconds)		70.37 ± 48.16	72.62 ± 25.19	126.95 ± 138.24	1.985
		(23–203)	(40–115)	(8–535)	
Clock Drawing Test Clicked erase		1.53 ± 2.29	0.83 ± 1.03	3.00 ± 4.69	1.689
		(0–8)	(0–3)	(0–16)	
Language					
Token Test	32.54 ± 3.16	33.04 ± 1.92	30.44 ± 3.58	30.50 ± 3.22	7.329***
	(20.5–36)	(28–36)	(21–35.5)	(22.5–36)	NC/SCI > MCI/D
BNT	12.30 ± 2.37	11.50 ± 3.35	10.50 ± 2.77	9.17 ± 2.21	19.326***
	(4–15)	(4–15)	(4–14)	(5–12)	NC > MCI/D, SCI > D

Summary statistics are presented as mean ± SD (standard deviation) and (range) for digital cognitive test results by final diagnostic group. Normative controls were calculated with the Mindmore normative models,^45,46^ with 100 virtual controls generated per participant (reported ranges reflect the valid scoring limits of each test). Group differences were evaluated using one-way ANOVAs with Tukey post hoc comparisons and presented as F and adjusted *p*-values. ***p < 0.001; **p < 0.01; *p < 0.05.

One Token Test result was invalid. Detailed Cube and Clock metrics were unavailable for 35 participants, as these measures were introduced part-way through the study; normative data are not yet available for these additional measures.

CERAD: Consortium to Establish a Registry for Alzheimer's Disease; SDPT: Symbol Digit Processing Test; TMT: Trail Making Test; BNT: Boston Naming Test; NC: normative controls; SCI: subjective cognitive impairment; MCI: mild cognitive impairment; D: dementia.

### Patient habits of digital tools in everyday life

The majority of patients reported daily smartphone use (n = 61, 80%), and nearly half reported daily computer use (n = 35, 46%). Tablet use was less common; almost half of the patients (n = 37, 49%) reported never using a tablet, and only 22% (n = 17) used one daily.

### Patient and clinician experiences of the digital cognitive assessment

Content analysis of patient responses to the question about differences between tablet-based and paper-based testing revealed six categories:

- Advantages of the digital format (29%), e.g., finding the digital tests more fun and creative, appreciating the interactive interface, and valuing the combination of written and audio instructions.
- No perceived difference (25%)
- Preference for paper-based tests (12%), e.g., greater ease in receiving instructions from a test leader, being able to ask questions, and writing with a pen on paper.
- More appropriate level of difficulty and greater comprehensiveness with the digital format (12%)
- Touchscreen interaction difficulties (5%)
- Increased stress with the digital format (5%)

Overall, patients appreciated the digital format, describing it as engaging and relevant, and 58% indicated they would prefer tablet-based testing for future assessments (Figure 2(a)).

**Figure 2.**
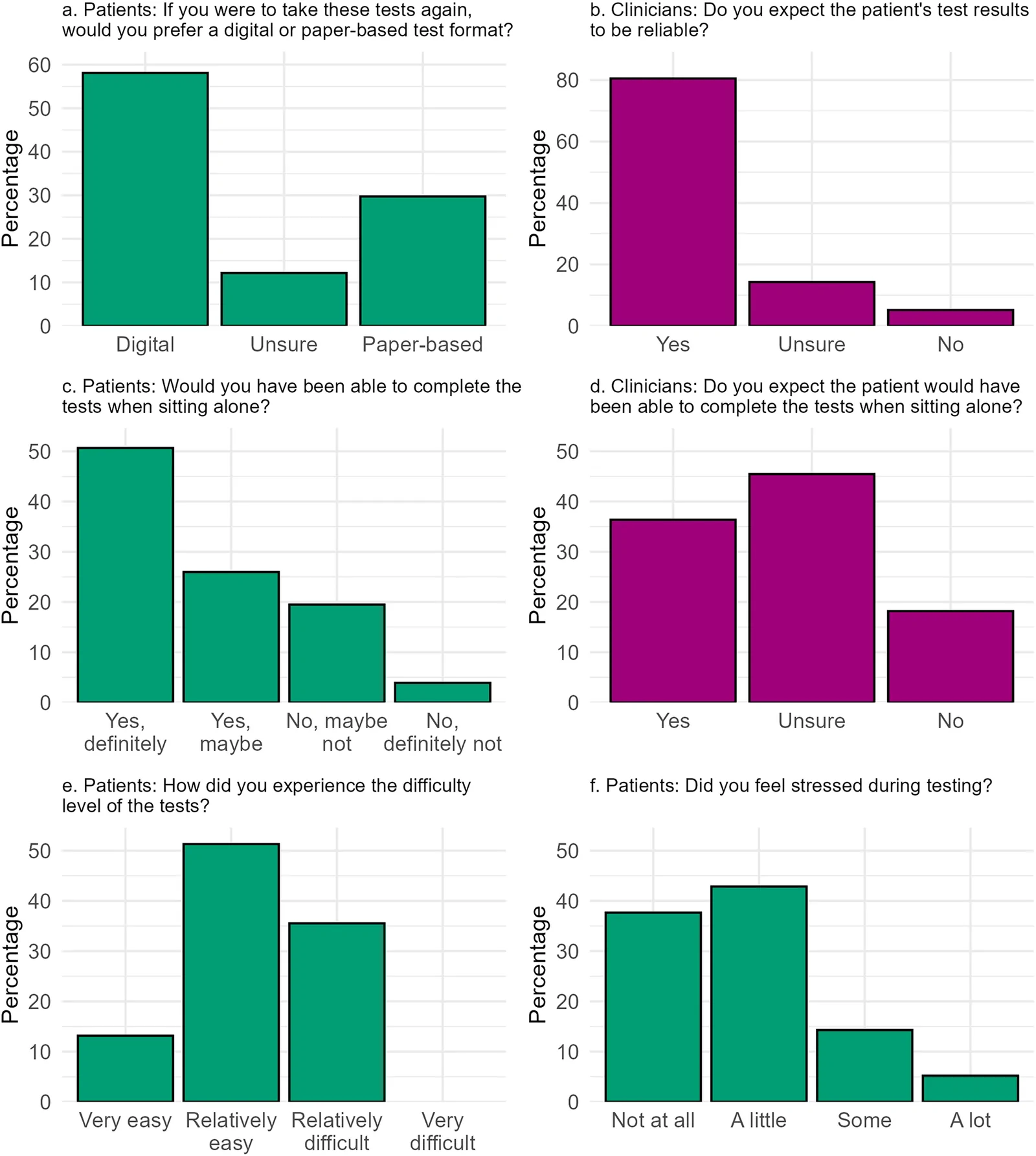
Patient and clinician experiences of the digital cognitive assessment. After each assessment, patients and occupational therapists (OTs) completed a hybrid survey. Results shown represent responses to the multiple-choice items. Percentages are based on patients or OTs responding to each item: n = 77 in panels b, c, d and f; n = 74 in panel a, where three patients did not select a response option, two of whom indicated in free text that either format would be acceptable; and n = 76 in panel e. No patient rated the difficulty level as ‘very difficult'.

OTs reported providing assistance in 69% of cases, and that 85% of patients asked one or more question. Types of assistance could be grouped into six categories:

- Supplementary test instructions (47%), typically repeating parts of the instructions when these were misunderstood or forgotten, or briefly pointing out interface elements such as where to press or how to interpret key symbols.
- Assistance with microphone use during verbal tasks (34%), including guidance on when to start speaking and how to interact with the microphone icon in the CERAD wordlist test.
- Touchscreen interaction difficulties (27%), such as the need to adjust finger pressure or repeat a touch when the first was not registered.
- Use of erase function in drawing tasks (22%), guidance on locating or activating the eraser tool in the Cube Copying and Clock Drawing tests.
- Guidance on test progression (18%), including reminding patients to press the “Next” button when moving within or between tasks.
- Affirming patients seeking confirmation (8%), offering brief reassurance, e.g., via eye contact or a nod, that instructions had been understood or that they could proceed.

Though needs for assistance were common, OTs rarely indicated concern that the results obtained under supervision would be compromised: 5% of cases were judged potentially unreliable (Figure 2(b)). Most patients (77%) believed they could complete the tests without supervision (Figure 2(c)); OTs indicated that 36% could likely complete the battery independently, whereas 18% could not (Figure 2(d)).

Patients rated the difficulty level as appropriate (Figure 2(e)), and several noted that the digital tasks felt more relevant to their cognitive abilities than brief paper-based screening tools such as the MMSE and CDT. OTs similarly did not consider any individual test systematically too difficult (as noted, TMT B floor effects are common in this population, and diagnostically informative); instead, they observed that different tests in the digital battery posed challenges for different patients. Patients did not find the digital assessment overly stressful (Figure 2(f)), a pattern corroborated by OT observations. Suggested improvements focused on clearer interface cues and optional stylus support.

## Discussion

This study suggests that a multidomain digital cognitive test battery is feasible, acceptable to both patients and OTs, and potentially usable within PHC neurocognitive assessments. Across cognitive domains, test performance broadly followed the expected gradient from SCI through MCI to dementia and provided a more detailed characterization of cognitive functioning compared to standard tools used in PHC. All included patients completed the test battery and regarded the tablet-based format as acceptable and often preferable to paper-based testing. OTs judged the digital tests appropriate for this population, provided some form of assistance in most cases, and anticipated the tests to yield reliable results. Together, these findings support the feasibility of integrating multidomain digital cognitive testing in PHC, with preliminary indications of clinical validity.

### Preliminary clinical validity

The strongest group-level differentiation emerged in the memory domain, consistent with the predominance of AD and mixed AD–vascular pathology in the dementia group. Performance on the CERAD word list learning, delayed recall, and recognition tasks differed between all diagnostic groups, in line with their well-established sensitivity to early and established neurocognitive disorders.^32,52^ These findings indicate that the core psychometric properties of these tests translate well to this digital format under supervised administration. With OT support available, usability issues such as overlooked interface cues did not appear to affect group differences.

Attention, processing speed, executive functions, and language tests also showed systematic group differences; both MCI and dementia groups differed significantly from SCI or normative controls in the SDPT, TMT B, Token Test, and BNT. In contrast, visuospatial performance was more variable. Cube Copying and Clock Drawing showed limited group-level differentiation, consistent with analog evidence from the present and other studies.^53,54^ The digital format provides additional performance indicators, time to completion and erasures, and video recordings which might provide further clinical value.

As expected from clinical experience, mixed results were observed for the standard PHC screening tools incorporated in diagnostic decision-making. MMSE and FAQ differentiated dementia from SCI and MCI, but not MCI from SCI, and CDT showed no significant post hoc group differences. This aligns with known limitations of brief screeners in detecting subtle or atypical impairment.^55,56^ As such, the multidomain digital cognitive assessment provides a more detailed characterization than the standard PHC screening tools used in this sample.

### Feasibility, acceptability, and usability in primary care

All 77 included patients completed the test battery, with one excluded following recent hand surgery. As part of the design, 14 single tests, 12 TMT B, were omitted from test administration after repeated failure on the practice trial. Since they were centered on TMT B and not TMT A, they likely indicated well-known effects of genuine cognitive impairment rather than feasibility issues.^51^ The median administration time was just under 45 min, substantially longer than for brief screening instruments such as the MMSE, though not unexpected for the detailed test battery used here, covering five cognitive domains with eight traditional tests yielding ten main outcomes. This positions the battery between brief screening instruments and comprehensive neuropsychological evaluation, which typically requires several hours of specialist administration.^57^ This time was borne by patients and supervising OTs rather than by the GP. Scoring and presentation of demographically corrected standardized scores were automated, so that in routine use the cognitive profile would be available to the clinician without further administration or scoring effort.

Both patients and OTs expressed positive views of the digital tests, consistent with earlier usability findings for Mindmore^42–44^ and generally favorable attitudes towards digital assessments in PHC.^17,21^ Patients in this study mostly regarded the tablet-based tests as acceptable and appropriately challenging, with many stating they would prefer them over paper-based testing for future assessments. OTs indicated that supervised digital administration did not compromise assessment quality and that supervision was most likely not necessary in more than a third of the sample, in spite of the comprehensiveness of the test battery and the level of cognitive impairment represented.

OTs did provide support in two-thirds of the test sessions, typically brief assistance such as clarifying instructions or pointing out interface elements, such as the erase buttons in the Cube and Clock Tests. These support needs may be partly resolvable through interface refinements, while some will likely persist in a patient population including individuals with substantial cognitive impairment. This pattern is consistent with a scoping review of shorter, unsupervised digital cognitive tests in relatively healthy, preclinical, AD cohorts, where 15–30% of participants reported difficulties or requested technical support.^20^ This need for support even in healthier populations highlights challenges with remote digital testing, which needs to be taken seriously to ensure clinically valid results.

To summarize, we found the multidomain structure aligns well with the heterogeneous PHC population. The gradient across SCI, MCI and dementia suggests potential for detecting early cognitive impairment, while domain-specific information may support differentiation among common etiologies.^2^ As fluid biomarkers, including BBM for AD and FTD, are expected to gradually become accessible in PHC,^12,13^ cognitive tools that complement rather than duplicate biomarker information will become more important. Because BBM identify underlying pathology but not the symptomatic cause of complaints, multidomain cognitive assessment remains essential for interpreting biomarker findings, establishing clinically meaningful diagnoses, and assessing level and profile of cognitive impairment to guide treatment and support.

### Limitations and future directions

The sample size restricted analysis of distinct MCI and dementia subtypes and limited the assessment to preliminary clinical validity; larger cohorts are needed to determine the diagnostic accuracy of individual tests and longitudinal data are warranted to investigate how the tests behave during disease progression. Comparisons involving the model-based normative control group warrant additional caution, as its large size may have inflated their statistical significance. Participants were asked to compare their experience of the digital assessment with comparatively briefer, less challenging paper-based testing (typically MMSE and CDT) completed earlier, which may have influenced their impressions. Mindmore interface refinements have been made since data collection, such that usability findings may not reflect the current platform version.

As a next step, studies examining how clinicians incorporate results from a multidomain digital cognitive test battery into diagnostic reasoning, referral decisions, and ongoing management would further clarify clinical utility. Future work may also explore whether additional digital metrics, particularly for visuospatial tests, enhance sensitivity, and are useful in primary care and/or specialist care settings.

### Conclusion

This study suggests that a comprehensive digital cognitive test battery is feasible, acceptable, and potentially usable in the heterogeneous primary care population, providing standardized multidomain cognitive profiles with limited clinical resource requirements. The majority of patients in this study population needed support from professionals (OTs) during the testing. By offering more detailed information than brief screening tests, digital testing may assist clinicians in detecting early cognitive impairment, informing referral decisions, guiding support interventions, and supporting clinical follow-up. As diagnostic demands in primary care continue to rise and treatment options evolve, multidomain digital cognitive assessment represents a promising approach for strengthening neurocognitive evaluation and intervention in PHC. Finally, the test results may also facilitate a more informed dialogue on cognitive functioning between general practitioners in PHC and consultant geriatricians.

## Supplemental Material

sj-docx-1-alr-10.1177_25424823261492238 - Supplemental material for Feasibility of digital multidomain cognitive testing in mild cognitive impairment and dementia assessment in primary care Supplemental material, sj-docx-1-alr-10.1177_25424823261492238 for Feasibility of digital multidomain cognitive testing in mild cognitive impairment and dementia assessment in primary care by Wobbie Van den Hurk, Maria M. Johansson, Elisabet Classon, Anders Gustavsson, Katarina Nägga and Anna Segernäs in Journal of Alzheimer's Disease Reports

## Data Availability

The data that support the findings of this study are not publicly available due to ethical restrictions but are available from the corresponding author upon reasonable request.
