# Supplementary material for: Feasibility of digital multidomain cognitive testing in mild cognitive impairment and dementia assessment in primary care
Source: J Alzheimers Dis Rep. 2026 Oct 7;10:25424823261492238. doi: 10.1177/25424823261492238 (PMC13662342; doi:10.1177/25424823261492238)
Supplement: Supplementary file 1 [file sj-docx-1-alr-10.1177_25424823261492238.docx]

**Supplemental Material**

**Feasibility of digital multidomain cognitive testing in mild cognitive impairment and dementia assessment in primary care**

**Supplemental Methods**

*Digital cognitive tests: administration and scoring*

The digital cognitive assessment was administered using the Mindmore platform and consisted of digital adaptations of established neuropsychological tests covering memory, attention and processing speed, executive functioning, visuospatial functioning, and language (also see Methods 2.4). Tests were self-administered with standardised on-screen instructions presented in both written and audio format, with audio instructions repeatable on demand. Tests were administered in a fixed order: Trail Making Test (TMT) Part A → TMT Part B → CERAD Word List Learning Trials 1–3 → Cube Copying → Clock Drawing → CERAD Delayed Recall → CERAD Recognition → Symbol Digit Processing Test (SDPT) → Token Test → Boston Naming Test (BNT).

*Memory: CERAD Word List Learning Test*

Memory was assessed using the 10-word CERAD Word List Learning Test, comprising three learning trials, a delayed free recall after approximately five minutes, and a recognition trial in which the 10 target words were presented among 10 distractor words.^1^ Learning performance was scored as the number of correctly recalled target words over the three learning trials. Delayed recall was scored as the number of correctly recalled target words during free recall. Recognition performance was calculated as the number of correctly recognised targets minus the number of incorrectly endorsed distractors (i.e., false positives). Scoring was performed automatically by an algorithm and confirmed or corrected by a blinded and trained rater.

*Attention and processing speed: TMT Part A and SDPT*

Attention and processing speed were assessed using TMT Part A^2^ and the SDPT.^3^ In TMT Part A patients are required to trace the number 1 to 23 in sequential order with their finger – in this version errors are not corrected. Performance is scored as time to completion and number of correct connections (max. 24). The test has a maximum duration of 120 seconds. The SDPT is a digital adaptation of the Symbol Digit Modalities Test (SDMT^3^) that uses a different symbol set across administrations to minimise memorisation effects. During 90 seconds, patients are required to match symbols, presented one-by-one, to digits. SDPT performance was scored number of correct responses.

*Executive functioning: TMT Part B*

Executive functioning (mental flexibility) was assessed using TMT Part B.^2^ Here patients are required to trace numbers and letters (1-A-2-B-3-C etc.) with their finger (errors are not corrected). Performance was quantified as time to completion and number of correct connections (max. 24). The test has a maximum duration of 240 seconds.

*Visuospatial functioning: Cube Copying and Clock Drawing*

Visuospatial performance was assessed using the Cube Copying Test^4^ and the Clock Drawing Test.^5,6^ The Cube Copying Test was scored dichotomously (1 = complete and correctly rendered three-dimensional cube; 0 = otherwise). The Clock Drawing Test was scored on a 0–1 scale, with 0.5 points assigned for correctly placed numbers and 0.5 points assigned for correctly drawn hands. During both tests patients are instructed to draw on the screen with their index fingers and can use two erasure buttons: erase last strike and erase all. Drawings from both visuospatial tasks were evaluated centrally by a blinded and trained rater.

*Language: Token Test and Boston Naming Test*

Language was assessed using the full 36-item Token Test^7,8^ and the short 15-item Boston Naming Test (BNT).^9,10^ Token Test performance was scored as number of correctly executed commands, with half a point if item instructions needed repeating (maximum one repeat). BNT performance was scored as the number of correctly named items, automatically by an algorithm and confirmed or corrected by a blinded and trained rater.

*Practice trials, omissions, and handling of omitted tests*

TMT Part A, TMT Part B, and SDPT were preceded by a practice trial. The practice trial had to be completed successfully before the corresponding test could begin; otherwise, the test was omitted after a maximum of five attempts. For omitted tests, results were imputed using the minimum observed value for that test in the current dataset (as described in Methods and Results).

*Technical requirements*

The flexibility of the platform supports continuity of assessment across care levels, from initial PHC assessment through specialist referral and disease follow-up, without requiring a change of platform, format, or normative framework. It also supports administration of clinical questionnaires and can be deployed across different hardware formats and languages, allowing flexible integration into different care contexts.

**References**

1.	Morris JC, Heyman A, Mohs RC, et al. The Consortium to Establish a Registry for Alzheimer’s Disease (CERAD). Part I. Clinical and neuropsychological assessment of Alzheimer’s disease. *Neurology* 1989; 39: 1159–65.

2.	Army Individual Test Battery. *Manual of directions and scoring*. Washington, DC: War Department, Adjutant General’s Office, 1944.

3.	Smith A. *Symbol Digit Modalities Test (SDMT). Manual (revised)*. Los Angeles: Western Psychological Services, 1982.

4.	Necker LA. Observations on some remarkable optical phænomena seen in Switzerland; and on an optical phænomenon which occurs on viewing a figure of a crystal or geometrical solid. *Lond Edinb Dublin Philos Mag J Sci* 1832; 1: 329–337.

5.	Shulman KI, Shedletsky R and Silver IL. The challenge of time: Clock‐drawing and cognitive function in the elderly. *Int J Geriatr Psychiatry* 1986; 1: 135–140.

6.	Critchley M. *The parietal lobes*. New York: Hafner, 1953.

7.	Boller F and Vignolo LA. Latent sensory aphasia in hemisphere-damaged patients: an experimental study with the Token Test. *Brain* 1966; 89: 815–830.

8.	De Renzi E and Vignolo LAA. The token test: A sensitive test to detect receptive disturbances in aphasics. *Brain* 1962; 85: 665–678.

9.	Jørgensen K, Johannsen P and Vogel A. A Danish adaptation of the Boston Naming Test: preliminary norms for older adults and validity in mild Alzheimer’s disease. *Clin Neuropsychol* 2017; 31: 72–87.

10.	Kaplan EF, Goodglass H and Weintraub S. The Boston Naming Test (2nd ed). Philadelphia, Lea & Febiger, 1983.
